# Editorial: Integrative Analysis of Genome-Wide Association Studies and Single-Cell Sequencing Studies

**DOI:** 10.3389/fgene.2021.752057

**Published:** 2021-08-27

**Authors:** Shiquan Sun, Sheng Yang

**Affiliations:** ^1^School of Public Health, Health Science Center of Xi'an Jiaotong University, Xi'an, China; ^2^Department of Biostatistics, School of Public Health, Nanjing Medical University, Nanjing, China

**Keywords:** genome-wide association analysis, single-cell RNA sequencing, integrative analysis, Mendelian randomization, transcriptome association analysis

Genome-wide association studies (GWAS) have identified thousands of genetic loci that are significantly associated with complex traits and diseases status. However, the functions/roles of the majority (~90%) of these associations remain poorly understood. Systematic characterization of their function is challenging because the function of variants of most traits likely acts in a tissue or cell-type specific fashion. The recent advances of single-cell sequencing technologies that enable characterization of epigenetic, proteomic, and transcriptomic profiles at individual cell, providing an unprecedented opportunity, alongside computational challenges, to comprehensively understand the functions/roles of associations in complex traits within the cellular perspective.

Therefore, integrating known functional cell-type specific annotations (e.g., cell-type specific expression levels etc.) into GWAS can potentially prioritize functional genetic variants and improve the performance of genomic predictions. Although various integrative analysis methods have been developed for such analyses, there is a pressing need to develop computationally scalable tools for large-scale GWAS, such as UK Biobank, China Kadoorie Biobank and FINNGEN. To address this need, this Research Topic focuses on integrative analysis to highlight the interpretation of genome-wide associations by leveraging the recent advances in single-cell sequencing studies.

In this special issue, we accepted 9 manuscripts on genome-wide association studies and/or single-cell sequencing in both methodology development and data analysis. We summarized the main contribution of these studies as follows:

Li et al. developed multiple computational approaches to deconvolve the bulk transcriptome data from whole kidney tissue with lupus nephritis (LN) into immune cell type-specific fractions and revealed that intrarenal mononuclear phagocytes might be an adjunctive histology marker for forecasting LN onset and retarded remission induction, which may facilitate on treatment and monitoring of LN patients.

Xiao et al. performed transcriptome-wide association study (TWAS) analysis on amyotrophic lateral sclerosis (ALS) and applied summary data-based Cauchy Aggregation TWAS (SCAT), a flexible *p*-value combination strategy, to integrate association signals from multiple brain tissues, and identified 5 new ALS-associated genes. Extensive simulations demonstrated that the proposed method can produce well-calibrated *p*-value for the control of type I error and more powerful to identify trait-association signals against single-tissue TWAS analysis.

Chen et al. performed genetic correlation analysis, gene-based association analysis, and pleiotropy-informed informatics analysis with coronary artery disease (CAD) and chronic kidney disease (CKD) related GWAS summary data, and identified common genetic architectures between the CAD and CKD, which may help to understand of the molecular mechanisms underlying the comorbidity of both diseases.

Gong et al. performed an integrative analysis of TWAS and mRNA expression profiles for idiopathic pulmonary fibrosis (IPF), and identified multiple novel candidate genes, GO terms and pathways for IPF, which would potentially contribute to the understanding of the genetic mechanism of IPF.

He et al. developed a new computational tool, single cell mixed model score tests (scMMSTs), to identify differentially expressed (DE) genes in single cell RNA sequencing (scRNA-seq) data with zero-inflation using the generalized linear mixed model (GLMM). Both simulations and real data analysis indicated that scMMSTs have more powerful performance in defining DE genes of zero-inflated scRNA-seq data with batch effects compared with the existing methods.

Ye et al. performed an integrative analysis on several GWAS and scRNA-seq data from chronic liver diseases (CLD), and identified B cell and NK cell as potential HCC-related cell types, which may supply clues for understanding the pathogenesis of CLD from a new angle.

Liu et al. developed a new agglomerative nesting clustering method for phenotypic dimensionality reduction analysis (AGNEP), which integrates agglomerative nesting clustering algorithm (AGNES) and principal component analysis (PCA) to detect genetic associations between SNPs and multiple phenotypes in GWAS. With extensive simulations and real data applications, AGNES shows more powerful performance in statistical power, computing time, and the number of quantitative trait nucleotides (QTNs).

Zhang et al. developed a flexible and scalable mixed linear model (MLM)-based method, the fast multi-locus ridge regression (FastRR), for QTNs dissection in GWAS. With simulations and real data applications, the results showed that the FastRR is more powerful for both large and small QTN detection, more accurate in QTN effect estimation, and has more stable results under various polygenic backgrounds.

Wang et al. developed a new deep convolutional neural network (CNN) of residual neural network (ResNet) on the whole-slide pathology features of breast cancer H&E stains and the patients' gBRCA mutation status, and the results demonstrated that the proposed method largely improve the prediction accuracy, which may potentially improve the cancer prognosis and therapeutics by utilizing biological markers currently imperceptible to clinicians.

With the further development of omics techniques and related analytical methods, integrative analysis on multiple omics data from the perspective of all in one will help to comprehensively understand the mechanism of complex traits and diseases status in large extent.

## Author Contributions

SS and SY wrote the editorial. Both authors have approved the submission.

## Conflict of Interest

The authors declare that the research was conducted in the absence of any commercial or financial relationships that could be construed as a potential conflict of interest.

## Publisher's Note

All claims expressed in this article are solely those of the authors and do not necessarily represent those of their affiliated organizations, or those of the publisher, the editors and the reviewers. Any product that may be evaluated in this article, or claim that may be made by its manufacturer, is not guaranteed or endorsed by the publisher.

